# The impact of tube replacement timing during LCIG therapy on PEG-J associated adverse events: a retrospective multicenter observational study

**DOI:** 10.1186/s12883-021-02269-7

**Published:** 2021-06-25

**Authors:** Kanefumi Yamashita, Yukinori Yube, Yukinao Yamazaki, Takehide Fukuchi, Masaki Kato, Tomoyuki Koike, Takeshi Uehara, Yoshiou Ikeda, Satoshi Furune, Hidehiro Murakami, Eiji Kubota, Shinsuke Fujioka, Yoshinori Sato, Xiaoyi Jin, Tomohiko Suzuki, Kazuhiro Furukawa, Yoshio Tsuboi

**Affiliations:** 1 Department of Gastroenterological Surgery, Seizan-Kai Kawaminami Hospital, Kawaminami-cho, Kawaminami 18150-47, Koyu-gun, Miyazaki 889-1301 Japan; 2 Department of Gastroenterology and Minimally Invasive Surgery, Juntendo University Hospital, Tokyo, Japan; 3 Department of Gastroenterology, Fukui Red Cross Hospital, Fukui, Japan; 4 Division of Endoscopy, Yokohama City University Medical Center, Kanagawa, Japan; 5 Division of Gastroenterology and Hepatology, Department of Internal Medicine, St Marianna University School of Medicine, Kanagawa, Japan; 6 Division of Gastroenterology, Tohoku University Graduate School of Medicine, Miyagi, Japan; 7 Department of Gastroenterology, Saitama Medical Center, Jichi Medical University, Saitama, Japan; 8 Department of Gastroenterology and Metabology, Ehime University Graduate School of Medicine, Ehime, Japan; 9 Department of Gastroenterology and Hepatology, Nagoya University Graduate School of Medicine, Nagoya, Japan; 10 Department of Internal Medicine, Saiseikai Matsuyama Hospital, Ehime, Japan; 11 Department of Gastroenterology and Metabolism, Nagoya City University Graduate School of Medical Sciences, Nagoya, Japan; 12 Department of Neurology, Fukuoka University, Fukuoka, Japan

**Keywords:** Levodopa–carbidopa intestinal gel, Parkinson’s disease, Percutaneous endoscopic gastrojejunostomy

## Abstract

**Background:**

Levodopa–carbidopa intestinal gel (LCIG) treatment, a unique drug delivery system for patients with advanced Parkinson’s disease (PD), is covered by health insurance in Japan since September 2016. Various LCIG procedure/device-associated adverse events (AEs) have been reported; however, reports on their treatment have been limited. This is the first multicenter study to clarify the frequency and timing of device-related AEs.

**Methods:**

Between September 2016 and December 2018, 104 patients introduced to the LCIG treatment for advanced PD in 11 hospitals were included. The patients’ characteristics, AEs incidence, AEs time, and tube exchange time were investigated.

**Results:**

The median follow-up period was 21.5 months. Minor AE cases were 29.4%, whereas major AE cases were 43.1%. Majority of major AEs (*n* = 55, 94.8%) were managed with endoscopic treatment, such as tube exchange. Few severe AEs required surgical treatment (*n* =3, 5.2%). The mean (range) exposure to percutaneous endoscopic gastrojejunostomy (PEG-J) was 14.7 (0–33) months. One year after the LCIG treatment introduction, 55 patients (54.0%) retained the original PEG-J tube. The mean PEG-J tube exchange time was 10.8 ± 7.0 months in all patients, 11.6 ± 4.7 and 10.5 ± 7.7 months in patients with scheduled exchange and who underwent exchange due to AEs, respectively.

**Conclusions:**

Some device-related AEs occurred during the LCIG treatment; however, only few were serious, most of which could be treated with simple procedures or tube replacement with endoscopy. Therefore, the LCIG treatment is feasible and safe and is a unique treatment option for PD, requiring endoscopists’ understanding and cooperation.

**Supplementary Information:**

The online version contains supplementary material available at 10.1186/s12883-021-02269-7.

## Background

As the risk of development of Parkinson’s disease (PD) sharply increases with age and due to the world’s aging population, the number of individuals affected is poised for exponential growth [1]. Although levodopa is the most effective treatment for PD, the long-term use of standard oral administration is associated with the development of motor complications such as dyskinesias and “on/off” fluctuations that can often be problematic with advancing disease [2–6]. However, maintaining therapeutic plasma concentrations of levodopa after an oral levodopa administration in patients with advanced PD is difficult due to the narrow therapeutic range and delayed gastric emptying [7, 8]. Levodopa–carbidopa intestinal gel (LCIG) treatment, a unique drug delivery system for patients with advanced PD, is a carboxymethylcellulose aqueous gel delivered directly to the proximal jejunum via a percutaneous endoscopic gastrojejunostomy (PEG-J) tube connected to a portable infusion pump. LCIG helps maintain higher therapeutic plasma concentrations of levodopa than standard oral levodopa therapy [4, 9]. It has been covered by insurance in Japan since September 2016. Global long-term study shows the safety and efficacy of LCIG infusion in advanced PD [10]. However, the LCIG administration cannot be achieved without PEG-J, which is the main hindrance to its introduction. Various LCIG procedure-/device-associated adverse events (AEs) have been reported, but not much is known about how to deal with them. In addition, LCIG treatment requires periodic tube replacement, but one of the problems is that there is no clear standard regarding the timing of tube replacement. Therefore, this study aimed to investigate the timing of tube replacement and the frequency and timing of device-related AEs and to clarify the appropriate timing for tube replacement. This is the first multicenter study on the frequency of LCIG procedure/device-associated AEs and the timing of tube replacement in Japan.

## Methods

This present multicenter retrospective study was approved by the institutional review board of the Fukuoka University School of Medicine (approval no.: 19–6-03) and all hospitals and was conducted according to the principles of the Declaration of Helsinki.

All 104 consecutive patients with advanced PD introduced to LCIG treatment in 11 hospitals between September 2016 and December 2018 were retrospectively examined.

The clinical data of advanced PD patients were retrospectively collected from the hospital medical records. The following clinical data were collected: age, sex, height, weight, duration of symptom, nasojejunal (NJ) tube/PEG-J tube placement method, gastrostomy method, NJ/PEG-J tube placement position, success rate of the procedure, procedure time, postoperative hospital days, follow-up period, PEG-J tube replacement timing, and device-related AEs. Device-related AEs were defined as cases that required any tube adjustment or replacement, deficiency of a portable infusion pump, breakage of percutaneous endoscopic gastrostomy (PEG)/PEG-J tube, any symptoms related to PEG/PEG-J tube placement, skin symptom around PEG. Device-related AEs were graded according to the Clavien–Dindo Classification [11] (Supplementary Table 1). Minor AEs were defined under Clavien–Dindo II and major AEs were defined over Clavien–Dindo IIIa.

The primary end point of the present study was the clarification of the frequency and timing of device-related AEs. The secondary end point of the present study was the clarification of the timing of tube replacement.

### LCIG treatment system

The NJ and PEG-J procedures were performed by an experienced gastroenterologist, radiologist, or surgeon. All proceduralists took training program course for LCIG treatment system.

Initially, to test the impact of LCIG on a patient, LCIG was administered via the NJ tube (Bengmark Nutricia Ch10, Cook NJFT-10) for 2–7 days. If the patient responded favorably, the PEG transabdominal tube (15 Fr FREKA) was inserted in the patient using the pull technique and PEG-J delivery system was introduced for long-term LCIG administration. PEG-J tube (9 Fr J-extension) was placed via the PEG tube under endoscopic guidance.

### Tube replacement

With regard to tube replacement, it was up to the clinician to decide whether to replace the tube after an AE occurred or at a certain period scheduled in advance. When AEs occurred, the neurologist or gastroenterologist decided whether tube replacement was necessary.

## Results

A total of 104 patients who underwent LCIG treatment for advanced PD were included in this study. The median follow-up period was 21.5 months. Clinical characteristics of all patients are summarized in Table 1. 48 were men and 56 were women. The mean age of patients was 66.9 ± 9.2 years and mean duration of PD was 72.7 ± 77.2 months.

**Table 1:** Characteristics of study population (*n* = 104)

Age (years), mean ± SD	66.9 ± 9.2
Sex, Male:Female, n (%)	48 (46.2):56 (53.8)
Weight (kg), mean ± SD	51.2 ± 11.4
Body mass index (kg/m^2^), mean ± SD	20.4 ± 3.6
Duration of Parkinson’s disease (months), mean ± SD	72.7 ± 77.2

### Technical details of the NJ tube and PEG-J tube placement

Insertion of the distal end of the NJ tube beyond ligament of Treitz was performed by endoscopic insertion while gripping the distal end of the intestinal tube with grasping forceps in eight hospitals, by endoscopic insertion or interventional radiologic insertion under fluoroscopic guidance in two hospitals, and by interventional radiologic insertion under fluoroscopic guidance in one hospital.

In all the hospitals, insertion of the distal end of the PEG-J tube beyond the ligament of Treitz was performed by endoscopic insertion while gripping the distal end of the intestinal tube using grasping forceps.

A total of 96 patients underwent NJ tube placement before PEG. The placement position of the NJ tube was the stomach in 4 (4.2%), descending duodenum in 3 (3.1%), horizontal duodenum in 14 (14.6%), and jejunum in 75 (78.1%) patients. The average operative time for NJ tube placement was 27.9 ± 19.4 min, as shown in Table 2.

**Table 2:** Characteristics of nasojejunal (NJ) tube placement (*n* = 96)

Placement position, n (%)	
Stomach	4 (4.2%)
Descending duodenum	3 (3.1%)
Horizontal duodenum	14 (14.6%)
Jejunum	75 (78.1%)
Success rate, n (%)	93 (96.9%)
Operation time (min), mean ± SD, range	27.9 ± 19.4 (8–132)
NJ skip, n (%)	8 (8.3%)
Drop out, n (%)	2 (2.1%)

Additionally, 102 patients underwent PEG and PEG-J tube placement. Among them, eight underwent PEG and PEG-J tube placement without NJ tube placement. Two patients dropped out of LCIG treatment after NJ tube placement because they refused further treatment. The placement position of the PEG-J tube was descending duodenum in 4 (3.9%), horizontal duodenum in 19 (18.6%), and jejunum in 79 (77.5%) patients. Gastropexy was performed in 91 patients (89.2%). Average operation time for PEG-J tube placement is 39.0 ± 21.6 min. PEG and PEG-J tube placement were successful in all cases, as shown in Tables 2 and 3.

**Table 3:** Characteristics of percutaneous endoscopic gastrojejunostomy tube placement (*n* = 102)

Placement position, n (%)	
Stomach	0
Descending duodenum	4 (3.9%)
Horizontal duodenum	19 (18.6%)
Jejunum	79 (77.5%)
Success rate, n (%)	102 (100%)
Gastropexy, n (%)	91 (89.2%)
Operation time (min), mean ± SD, range	39.0 ± 21.6 (14–120)

During the LCIG treatment, 74 (72.5%) of the 102 patients experienced at least one AE. Among those AEs, immediate AEs, which were occurred within a month, were 11 cases (10.5%) and delayed AEs, which were occurred after one month, were 94 cases (89.5%).

Major AEs were observed in 58 patients (55.2%), including tube dislocation (*n* = 27, 25.7%), tube breakage (*n* = 12, 11.4%), tube occlusion (*n* = 7, 6.7%), tube kink (*n* = 3, 2.9%), connector trouble (*n* = 3, 2.9%), peritonitis (*n* = 3, 2.9%), bezoar (*n* = 2, 1.9%), and postoperative wound infection (*n* = 1, 1.0%).

Minor AEs were observed in 47 patients (44.8%), including tube occlusion (*n* = 10, 9.5%), postoperative wound infection (*n* = 7, 6.7%), wound granulation (*n* = 7, 6.7%), tube dislocation (*n* = 6, 5.7%), tube kink (*n* = 5, 4.8%), connector trouble (*n* = 4, 3.8%), tube breakage (*n* = 3, 2.9%), pump trouble (*n* = 2, 1.9%), and others including aspiration pneumonia (*n* = 3, 2.9%) (Table 4).

**Table 4:** Incidence of procedure-/device-associated minor adverse events (AEs) and major AEs

	n (%)
All cases	102 (100)
Cases with AEs	74 (72.5)
All AEs	105 (100)
Immediate AEs	11 (10.5)
Delayed AEs	94 (89.5)
Major AEs	58 (55.2)
Tube dislocation	27 (25.7)
Tube breakage	12(11.4)
Tube occlusion	7(6.7)
Tube kinking	3(2.9)
Connector trouble	3(2.9)
Peritonitis	3 (2.9)
Bezoar	2(1.9)
Postoperative wound infection	1 (1.0)
Minor AEs, n (%)	47 (44.8)
Tube occlusion	10 (9.5)
Postoperative wound infection	7 (6.7)
Wound granulation	7 (6.7)
Tube dislocation	6(5.7)
Tube kinking	5 (4.8)
Connector trouble	4(3.8)
Tube breakage	3 (2.9)
Pump trouble	2 (1.9)
Others	3 (2.9)

Regarding management of AEs, most of the major AEs (*n* = 55, 94.8%) were managed by endoscopic treatment such as tube exchange. Only few cases showed severe AEs that required surgical treatment (*n* = 3, 5.2%) (Table 5).

**Table 5:** Major adverse events (AEs) and their management

	n (%)	Treatment type (Endoscopy or Surgery)
Major AEs	58 (100)	
Tube dislocation	27 (46.6)	27 cases: Endoscopic treatment
Tube breakage	12 (20.7)	12 cases: Endoscopic treatment
Tube occlusion	7(12.1)	7 cases: Endoscopic treatment
Tube kinking	3 (5.2)	3 cases: Endoscopic treatment
Connector trouble	3(5.2)	3 cases: Endoscopic treatment
Peritonitis	3 (5.2)	3 cases: Surgery
Bezoar	2(3.4)	2 cases: Endoscopic treatment
Postoperative wound infection	1 (1.7)	1 case: Endoscopic treatment

The mean time to the onset of procedure/device-associated first AEs was 231.0 ± 202.4 days in all patients with AEs, 145.3 ± 119.8 days in minor AEs, and 271.9 ± 220.2 days in major AEs (Table 6).

**Table 6:** Time to procedure-/device-associated first adverse events (AEs)

All cases	days, mean ± SD, median (range)	231.0 ± 202.4, 200 (0–934)
Immediate AEs	days, mean ± SD, median (range)	6.9 ± 7.4, 4 (0–26)
Delayed AEs	days, mean ± SD, median (range)	274.2 ± 193.2, 224 (32–934)
Minor AEs	days, mean ± SD, median (range)	145.3 ± 119.8, 109.5 (2–441)
Major AEs	days, mean ± SD, median (range)	271.9 ± 220.2, 231 (0–934)

**Fig. 1:**
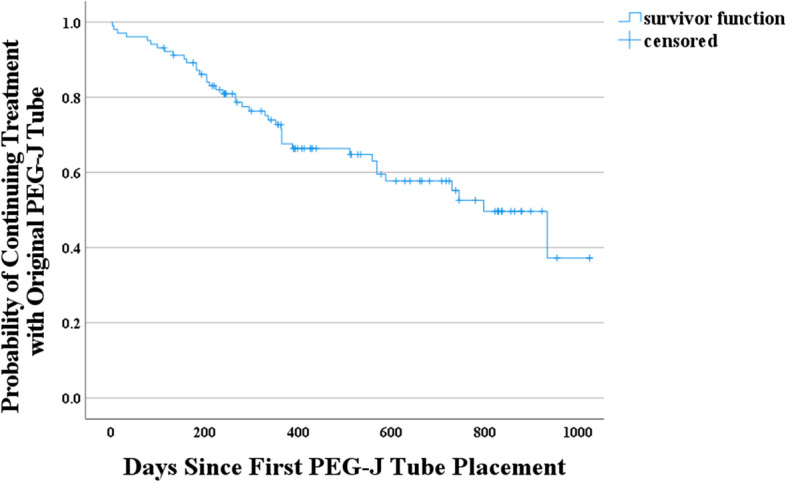
Retention period of the original PEG-J tube (All PEG-J, *N* = 102). PEG-J = percutaneous endoscopic gastrojejunostomy, All PEG-J = dataset of patients who had PEG-J tube placement

The mean (range) time of LCIG treatment exposure was 14.8 (0–33) months. At 1 year after the introduction of LCIG treatment, 56 patients (54.9%) retained the original PEG-J tube with a cumulative survival rate (SD) of 0.68 (0.05). At 18 months, 37 patients (36.3%) retained the original PEG-J tube with a cumulative survival rate (SD) of 0.63 (0.05). At 2 years, only 23 patients (22.5%) retained the original PEG-J tube with a cumulative survival rate (SD) of 0.55 (0.06) (Fig. 1; Table 7).

**Table 7:** Exposure to original percutaneous endoscopic gastrojejunostomy (PEG-J) tube

Duration	All PEG-J (*N* = 102), n (%)
≥ 1 year	56 (54.9)
≥ 18 months	37 (36.3)
≥ 2 years	23 (22.5)
Mean ± SD (months)	14.75 ± 8.62
Median (range) (months)	12.28 (0–33)

At 1 year after the introduction of LCIG treatment, 54 patients (65.1%) retained the original PEG-J tube in patients with follow-up for > 1 year without scheduled PEG-J tube replacement. At 18 months, 36 (43.3%) of these patients retained the original PEG-J tube. At 2 years, 22 patients (26.5%) retained the original PEG-J tube (Supplementary Table 2).

The mean time to PEG-J tube exchange was 10.4 ± 6.8 months in all the cases, 11.0 ± 4.5 months in the scheduled exchange cases, and 10.2 ± 7.4 months in the cases of exchange for AEs (Table 8). Details about the exchange for patients with AEs are shown in Table 4.

**Table 8:** Time and reason for percutaneous endoscopic gastrojejunostomy tube exchange

All cases, n (%)	55
Months, mean ± SD, median (range)	10.4 ± 6.8, 8.7 (0–31)
Scheduled exchange, n (%)	15
Months, mean ± SD, median (range)	11.0 ± 4.5, 11.7 (4–22)
Exchange for AE, n (%)	40
Months, mean ± SD, median (range)	10.2 ± 7.4, 9.0 (0–31)

## Discussion

This is a large retrospective multicenter study conducted in 11 hospitals to investigate the frequency of device-related AEs and AEs associated with PEG-J tube replacement timing during LCIG treatment. In this study, PEG-J was successfully and safely inserted in all patients.

However, a high rate of AEs was reported in not only PEG-J insertion for enteral nutrition [12–16] but also for LCIG treatment [10, 17–20]. Furthermore, studies describing AE details and ways to deal with them are limited. Therefore, this study clarifies details of LCIG-related AEs, including the frequency and timing of their occurrence and ways to manage them. Several studies have reported that the incidence was 47.2%–87.5% for any AEs related to the LCIG treatment and 17%–44% for major AEs related the LCIG treatment [10, 17–20]. On the contrary, in this study, 29.4% (*n* = 30) of patients had minor and 43.1% (*n* = 44) had major AEs as in previous reports. In previous reports, the definitions of major and minor AEs were vague. We used the Clavien–Dindo classification to classify AEs. As per this classification, any case with an AE requiring tube replacement is classified as a major complication because tube replacement using endoscopy or fluoroscopy is classified as ≥ grade IIIa. For this reason, major complications may appear to be more prevalent in this study.

The incidence rate of AEs in all patients was 72.5% (*n* = 74). The incidence rate of AEs related to tube problems such as tube dislocation, tube kinking, tube occlusion, and tube breakage was 69.5% (*n* = 73), of which 32.9% (*n* = 24) were treated with vigorous saline flushing and/or declogging of the PEG-J tube with an inserted guidewire agitated back and forth. The remaining (67.1%, *n* = 49 cases) AEs related to tube problems were treated with PEG-J tube exchange using endoscopy or fluoroscopy. Other studies demonstrated that most of the procedure/device-related AEs reported by patients who had a PEG-J placement in these studies were consistent in nature and incidence, with medically recognized AEs of the procedure in non-PD patient populations [10, 17–25].

In our study, major AEs were observed in 43.1% (*n* = 44) of all patients. Among these events, only few were severe and required surgical treatment and most major AEs were treated endoscopically such as with tube exchange. In two cases with severe AE, surgical intervention was necessary because of peritonitis, which was probably due to PEG. In one case, cholecystitis occurred immediately after PEG, requiring surgical intervention, although the relationship between cholecystitis and PEG was unclear. In all the cases, the general condition improved after surgery, and LCIG treatment could be continued.

Therefore, we can say that only 2.9% (*n* = 3 cases) of the AEs were notable serious AEs and the remaining 97.3% of the AEs could be treated with simple procedures such as vigorous saline flushing or endoscopic tube exchange in this study. Serious AEs occurred in 2.9% (*n* = 3 cases) of all the cases, which indicates that the LCIG treatment was safe and feasible.

The bezoar formation can be a problem during LCIG treatment due to the need for long-term placement of PEG-J tubes. AEs with bezoar, which is a mass of indigestible ingested particles accumulated over time in the gastrointestinal system, were reported in patients who received LCIG treatment [26]. Bezoar can cause pressure ulcers, obstruction of the gastrointestinal tract, or difficulty to remove the PEG-J tube due to adhesion of the bezoar to the intestinal wall. In fact, two cases of bezoar were observed in this study. In these two patients, bezoar was formed at the tip of the tube. The tip of the PEG-J tube of the LCIG treatment system is pigtail-shaped. This shape may easily trap food residues, causing a bezoar. Especially, Japanese foods contain a large amount of non-digestible fibers such as soybeans, sweet potato, burdock, cabbage, mushrooms, and seaweed. A bezoar is composed of these non-digestible fibers [26, 27]. This is the reason why Japanese people should be careful of bezoar formation during LCIG treatment. Probably, the best way to prevent this complication is to maintain a low-fiber diet. [26] Especially in cases with long-term PEG-J tube placement such as in LCIG treatment, attention should be paid to the possibility of bezoar formation, and patients receiving LCIG treatment should be advised to avoid fiber-rich diets.

At the end of the first year, 54.9% of patients retained the original PEG-J tube, and at the end of the second year, 22.5% retained the original PEG-J tube.

The original PEG-J tube retention rates reported in other studies were 63 and 49% at the end of the first and second years, respectively, and the PEG-J tube retention rate in the present study tended to be shorter than those in other studies [17, 20]. However, even in this study, the PEG-J tube retention rate after 1 year was 65.1% in the analysis, excluding patients with scheduled replacement. In Japan, this may have affected the tube retention rate because some facilities regularly exchange tubes even without AEs.

A total of 53 patients had their tubes replaced one or more times. The mean replacement period was 10.4 months, i.e., 11.0 months for patients with scheduled replacement and 10.2 months for patients who had to undergo replacement due to AEs. No statistically significant difference was observed in terms of retention periods to PEG-J tubes between the two groups. In this study, 65% of AEs requiring tube replacement occurred within one year. However, the time of occurrence of AEs requiring tube replacement varied (Supplementary Table 3). Considering that the general PEG-J tube for enteral nutrition (non-LCIG PEG-J tube) is replaced every 6 months, the average duration of replacement in patients with tube replacement due to AEs was approximately 10 months, and 65% of AEs requiring replacement occurred within one year. Hence, the need for replacing the LCIG PEG-J tube may be necessary in the first year. Additionally, the PEG-J tube should be checked every 6 months for patency, residue adhesion, among others, for the early detection of AEs.

In this study, we also observed many tube-related AEs, but most of them could be treated with simple procedures or endoscopic tube replacement. No serious AEs caused by long-term tube placement were observed in this study. Most of the AEs could be managed with tube replacement. If patients are given enough information, they may choose to replace the tube when AEs requiring tube replacement occur, without periodic tube replacement.

At present, the timing of tube replacement should be handled flexibly depending on the situation of each facility or patient. In Japan, new tubes that can be easily replaced are now available. Further research with a long-term observation period is desirable.

## Conclusions

In conclusion, as in previous reports, some device-related AEs occurred during the LCIG treatment, but only few of them were serious, most of which could be treated with simple procedures or tube replacement using endoscopy. Therefore, the LCIG treatment is feasible and safe to perform. However, careful observation of the PEG-J tube is required because LCIG treatment requires long-term tube placement.

The LCIG treatment system is a unique and useful treatment option for PD and requires the understanding and cooperation of endoscopists.

## Supplementary Information

**Additional file 1: Supplementary Table 1.** The Clavien–Dindo classification. **Supplementary Table 2.** Exposure to original percutaneous endoscopic gastrojejunostomy (PEG-J) tube of cases with follow-up for more than 1 year. **Supplementary Table 3.** Time to first major adverse events (AEs).

## Data Availability

The datasets used and analyzed in this study are available from the corresponding author on reasonable request.

## Abbreviations

PD: Parkinson’s disease
LCIG: Levodopa–carbidopa intestinal gel
PEG-J: Percutaneous endoscopic gastrojejunostomy
AE: Adverse event
PEG: Percutaneous endoscopic gastrostomy
