# Supplementary material for: The impact of tube replacement timing during LCIG therapy on PEG-J associated adverse events: a retrospective multicenter observational study
Source: BMC Neurol. 2021 Jun 25;21:242. doi: 10.1186/s12883-021-02269-7 (PMC8228941; doi:10.1186/s12883-021-02269-7)
Supplement: Supplementary file 1 — Additional file 1: Supplementary Table 1. The Clavien–Dindo classification. Supplementary Table 2. Exposure to original percutaneous endoscopic gastrojejunostomy (PEG-J) tube of cases with follow-up for more than 1 year. Supplementary Table 3. Time to first major adverse events (AEs). [file 12883_2021_2269_MOESM1_ESM.docx]

The impact of tube replacement timing during LCIG therapy on PEG-J associated adverse events: a retrospective multicenter observational study

Kanefumi Yamashita^1^, Yukinori Yube^2^, Yukinao Yamazaki^3^, Takehide Fukuchi^4^, Masaki Kato^5^, Tomoyuki Koike^6^, Takeshi Uehara^7^, Yoshiou Ikeda^8^, Satoshi Furune^9^, Hidehiro Murakami^10^, Eiji Kubota^11^, Shinsuke Fujioka^12^, Yoshinori Sato^5^, Xiaoyi Jin^6^, Tomohiko Suzuki^9^, Kazuhiro Furukawa^9^, Yoshio Tsuboi^12^

^1^Department of Gastroenterological Surgery, Seizan-Kai Kawaminami Hospital, Miyazaki, ^2^Department of Gastroenterology and Minimally Invasive Surgery, Juntendo University Hospital, Tokyo, ^3^Department of Gastroenterology Fukui Red Cross Hospital, Fukui, ^4^Division of Endoscopy, Yokohama City University Medical Center, Kanagawa, ^5^Division of Gastroenterology and Hepatology, Department of Internal Medicine, St Marianna University School of Medicine, Kanagawa, ^6^Division of Gastroenterology, Tohoku University Graduate School of Medicine, Miyagi, ^7^Department of Gastroenterology, Saitama Medical Center, Jichi medical University, Saitama, ^8^Department of Gastroenterology and Metabology, Ehime University Graduate School of Medicine, Ehime, ^9^Department of Gastroenterology and Hepatology, Nagoya University Graduate School of Medicine, Nagoya, ^10^Department of Internal Medicine, Saiseikai Matsuyama Hospital, Ehime, ^11^Department of Gastroenterology and Metabolism, Nagoya City University Graduate School of Medical Sciences, Nagoya, ^12^Department of Neurology, Fukuoka University, Fukuoka, Japan

**Supplementary Table**

| Supplementary Table 1. The Clavien–Dindo classification |  |  |  |  |  |  |  |  |  |  |  |
| --- | --- | --- | --- | --- | --- | --- | --- | --- | --- | --- | --- |

| **Grades** | **Definition** |  |  |  |  |  |  |  |  |  |  |  |  |  |  |
| --- | --- | --- | --- | --- | --- | --- | --- | --- | --- | --- | --- | --- | --- | --- | --- |
| Grade I | Any deviation from the normal postoperative course without the need for pharmacological treatment or surgical, endoscopic, and radiological interventions Allowed therapeutic regimens: drugs, such as antiemetics, antipyretics, analgesics, diuretics, and electrolytes, and physiotherapy. This grade also includes wound infections opened at bedside | | | | | | | | | | | | | | |
|  |  |  |  |  |  |  |  |  |  |  |  |  |  |  |  |
|  |  |  |  |  |  |  |  |  |  |  |  |  |  |  |  |
| Grade II | Requiring pharmacological treatment with drugs other than those allowed for grade I complications. | | | | | | | | |  |  |  |  |  |  |
|  | Includes blood transfusions and total parenteral nutrition | | | | | |  |  |  |  |  |  |  |  |  |
| Grade III | Requiring surgical, endoscopic, or radiological intervention | | | | |  |  |  |  |  |  |  |  |  |  |
| - IIIa | Intervention not under general anesthesia | | | |  |  |  |  |  |  |  |  |  |  |  |
| - IIIb | Intervention under general anesthesia | | | |  |  |  |  |  |  |  |  |  |  |  |
| Grade IV | Life-threatening complications requiring intermediate care/intensive care unit management | | | | | | | |  |  |  |  |  |  |  |
| - IVa | Single-organ dysfunction (including dialysis) | | | |  |  |  |  |  |  |  |  |  |  |  |
| - IVb | Multiorgan dysfunction | |  |  |  |  |  |  |  |  |  |  |  |  |  |
| Grade V | Death of the patient | |  |  |  |  |  |  |  |  |  |  |  |  |  |

| Supplementary Table 2. Exposure to original percutaneous endoscopic gastrojejunostomy (PEG-J) tube of cases with follow-up for more than 1 year | |
| --- | --- |
| Duration | All PEG-J (N = 83), n (%) |
| ≥1 year | 54 (65.1) |
| ≥18 months | 36 (43.3) |
| ≥2 years | 22 (26.5) |
| Mean ± SD (months) | 16.4 ± 9.0 |
| Median (range) (months) | 16.9 (0–33) |

| Supplementary Table 3. Time to first major adverse events (AEs) | |
| --- | --- |
| Duration | AEs cases (N = 40), n (%) |
| ≥6 months | 11 (27.5) |
| ≥1 year | 26 (65.0) |
| ≥18 months | 32 (80.0) |
| ≥2 years | 36 (90.0) |
